# Prediction of Bacillus Calmette-Guerin Response in Patients with Bladder Cancer after Transurethral Resection of Bladder Tumor by Using Genetic Variation Based on Genomic Studies

**DOI:** 10.1155/2016/9859021

**Published:** 2016-11-08

**Authors:** Ning Zhang, Guangliang Jiang, Xu Liu, Rong Na, Xiang Wang, Jianfeng Xu

**Affiliations:** ^1^Department of Urology, Huashan Hospital, Fudan University, Shanghai, China; ^2^Fudan Institute of Urology, Huashan Hospital, Fudan University, Shanghai, China; ^3^Program for Personalized Cancer Care, NorthShore University HealthSystem, Chicago, IL 60201, USA; ^4^Fudan Center for Genetic Epidemiology, School of Life Sciences, Fudan University, Shanghai, China; ^5^Department of Urology, Shanghai First People's Hospital, School of Medicine, Shanghai Jiao Tong University, Shanghai, China

## Abstract

*Purpose.* We aimed to comprehensively review contemporary literature on genetic and epigenetic biomarkers associated with the prediction of Bacillus Calmette-Guerin (BCG) response after the transurethral resection of a bladder tumor and to discuss the application of these biomarkers in precision cancer care for bladder cancer.* Method.* We performed a systematic review of published literatures in the databases PubMed and Embase by using the following key words: bladder cancer, BCG, gene, and methylation. Studies associated with cell lines, animal models, and muscle invasive bladder cancer were excluded.* Results.* The genetic variations associated with BCG response can be classified into three categories: germline variations, somatic variations, and epigenetic alterations. Genes related to BCG response were mainly involved in single-nucleotide polymorphisms, copy number variations, and gene methylations.* Conclusions.* Although these gene alterations are currently the most promising predictive markers of BCG response, most studies about bladder cancer DNA biomarkers are related to germline variations in candidate genes, and the results are not consistent. Only one study is related to somatic variation, and further evaluation in large-scale validation studies should be conducted to assess the potential clinical application of these findings. In addition, other biomarkers based on different “–omics” technologies should be considered in future studies.

## 1. Background

Bladder cancer (BC) is the most common malignancy of the urinary tract and the 7th most common cancer in men and the 17th in women [1]. Nearly 75% of bladder cancer patients are nonmuscle invasive bladder cancer (NMIBC), which can be treated with transurethral resection of bladder tumor (TURBT). Postoperative intravesical treatments are recommended based on clinical guidelines. For patients with intermediate-risk and high-risk tumors, intravesical full-dose Bacillus Calmette-Guerin (BCG) instillation is recommended as the first choice of treatment [2]. However, nearly 40% of patients do not respond to intravesical BCG therapy and ultimately experience disease recurrence or progression, which leads to a poorer prognosis [3, 4]. Due to the limitations of one-size-fits-all clinical treatment of bladder cancer using BCG, it is of clinical importance to predict individual response to BCG treatment and provide personalized treatment for these patients. For example, using mitomycin or other chemotherapy agents for bladder instillation in patients who do not response to BCG may improve the disease prognosis.

Genetic alterations have been reported to play important roles in BCG response. First, genetic alterations in single nucleotides in numerous pathways were found to be closely associated with BCG response. These alterations included single-nucleotide polymorphisms (SNPs), single-nucleotide variations (SNVs), and mutations [5, 6]. Second, various genetic copy number alterations were associated with bladder cancer [7]. Third, epigenetic regulation of genes, especially by gene methylation, was found to be associated with BCG failure for multiple genes [8–10].

There have been few systematic review papers associated with BCG response in the past 15 years and only one systematic review paper published in 2012 [11]. In this systematic review, we aimed to review the contemporary literature on biomarkers predicting BCG response, particularly in genetics and epigenetics.

## 2. Evidence Acquisition

**Figure 1:**
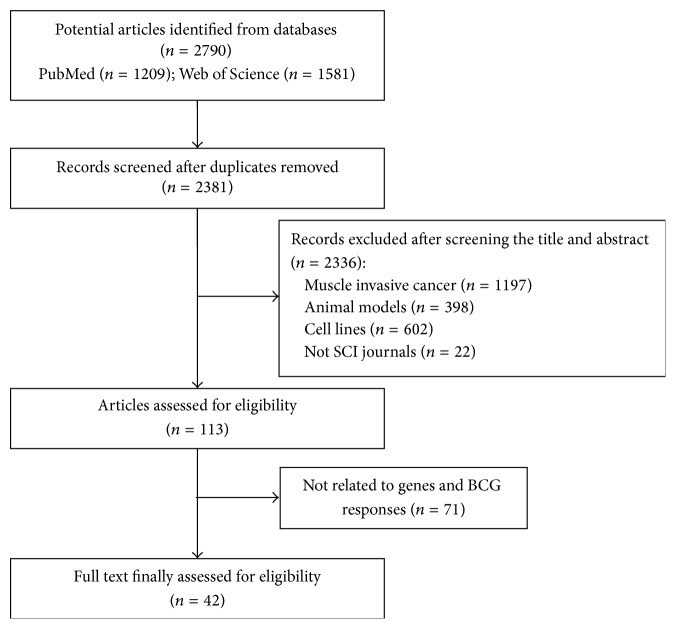
Flowchart of study selection process.

A systematic review of published literature based on population studies published during 2000–2016 in PubMed and Embase databases was performed. The following keywords were used: bladder cancer, BCG, gene, SNP, copy number, and methylation. Studies involved in cell lines, animal models, and muscle invasive bladder cancer were excluded. Published articles that did not belong to the science citation index were also excluded. Ultimately, 2790 articles were found based on the keywords, 42 articles of which that had full text available were reviewed in detail, and 22 of them were included based on exclusion criteria (Figure 1). All articles were examined by two authors, and papers related to our research purpose were selected.

## 3. Evidence Synthesis

### 3.1. Germline Variations

#### 3.1.1. Inflammatory Genes

The antitumor activities of BCG were related closely to immune system activation, so cytokines and immune cells played the key role in the process of antitumor. Therefore, polymorphisms of genes involved in the processes of BCG immunotherapy mechanism would affect the BCG response [12].

Single-nucleotide polymorphisms (SNPs) within the interleukin family were found to be associated with BCG response (Table 1). Leibovici et al. found that SNP-174(C/C) at* IL-6* was associated with an increased risk of disease recurrence (HR: 4.60) in patients receiving BCG after TURBT [13]. However, another study indicated that SNP-174(C/C) at* IL-6* was associated with a decreased risk of recurrence (HR = 0.298) and an increased recurrence-free survival (RFS) [14]. Studies on* TNF-a* polymorphisms reported that the SNP rs1799964(C/C) at* TNF-a* was associated with a decreased risk of recurrence [6, 15]. Ahirwar et al. found that rs4073 (at* IL-8*) was significantly associated with a decreased risk of recurrence (*p* < 0.001) and an increased RFS (*p* < 0.001) in patients who underwent BCG treatment after TURBT, but these results have not yet been confirmed [13]. Rs2070874 at* IL-4*, rs2104286 at* IL2RA*, and rs4819554 at* IL17RA* were also reported to be associated with disease recurrence after BCG treatment [6].

SNPs at other immune factor genes were also found to be related to BCG response. Studies showed that* TGF-B*(+28 T/T) was associated with a decreased risk of recurrence [16], while rs79037040(T/T) at* TRAILR1* and* IFN-G*(+874 A/A) and rs2104286(T/T) at* IL2RA* were associated with an elevated risk of recurrence after BCG immunotherapy [6, 16]. A germline deletion in the promoter area of* NF-kB* was associated with an elevated risk of recurrence after BCG immunotherapy [17].

Furthermore, one study reported a predictive score that conferred likelihood of responding to BCG treatment. In the study, BCG response-associated SNPs, together with gender, number of tumor, and treatment scheme, were included in the risk model. Patients were classified into risk groups based on risk score: patients with low risk had a 90% likelihood of successful treatment (no recurrence), while patients with high risk had only a 25% likelihood of success (75% recurrence) after BCG treatment. The AUC for this risk model was 0.82 [6]. This genetic risk classifier may be applied to personalized cancer care for patients before BCG treatment.

#### 3.1.2. Glutathione (GSH) Pathway Genes

GSH pathway is involved in cell detoxification and antioxidation and may be associated with oncogenesis, prognosis, and response to treatment [18, 19]. Therefore, genetic variants in the GSH pathway would influence cancer recurrence and response to BCG treatment after TURBT in NMIBC patients. Genetic variants in the GSH pathway may influence BCG response for patients after TURBT (Table 2).

A prospective study indicated that* hGPX1*(198 C/T) was associated with risk of recurrence of NMIBC during BCG immunotherapy [20]. Similar results were reported by Chiong et al. [21]. In several studies [22, 23], the polymorphisms of GSH pathway genes, for example,* GSS*(rs7265992),* GSS*(rs6060124),* GSS*(rs4911455),* GPX2*(rs10133290),* GSTM4*(rs560018), and* GSTT1*-positive, were significantly associated with increased risk of recurrence after BCG treatment. The polymorphisms of* GPX5*(rs377514) and* GSTM3*(rs4970737) had protective effects against recurrence after intravesical instillation BCG.

#### 3.1.3. Nucleotide Excision Repair (NER) Gene

NER is one of the major DNA repair pathways related to the removal of a wide variety of DNA lesions, and some studies have revealed that the polymorphisms of DNA repair genes might be risk factors for many tumors [24]. A possible mechanism is that SNPs in NER genes may alter the DNA repair process and affect clinical outcome of bladder cancer.

The studies showed that patients who carried SNPs or mutations, such as rs1800975(A/A, in* XPA*) and Met1097Val (A/A,* ERCC6*), would have longer RFS during BCG treatment [25], while carrying* ERCC2*(312 A/A) and Lys939Gln(C/C,* XPC*) was associated with high risk of recurrence and shorter RFS in BCG-treated patients [26, 27].

#### 3.1.4. Pathways Genes of Apoptosis and Proliferation

The FAS/FASL system plays an important role in cell apoptosis, proliferation, and immune system regulation. Deregulation of the FAS/FASL system may lead to immune escape of tumor cells, thereby influencing BCG immunotherapy outcomes [28]. Lima et al. demonstrated that carriers of the* FASL-844* C/C genotype presented an increased risk of BCG treatment failure and a decreased RFS after BCG treatment when compared with* FASL-844* T allele carriers (HR: 1.92, *p* = 0.03) [29]. This result was able to be validated in an independent study (HR: 1.71, *p* = 0.04) [6].

Survivin, the expression product of the* Survivin* gene, is a member of the novel inhibitor of apoptosis protein (IAP) family, which is expressed in human malignancy, but almost undetectable in normal or well-differentiated adult tissues [30]. A study in patients undergoing BCG therapy showed that rs9904341(C/C) at* Survivin* was significantly correlated (HR: 0.35, *p* = 0.009) with a reduced risk of bladder cancer recurrence and a better RFS. Therefore, variant genotype CC also showed a protective association with bladder cancer [31].

#### 3.1.5. Sonic Hedgehog (Shh) Pathway

Shh pathway is one of the major signal pathways that regulates cancer stem cells, cell proliferation, and differentiation, which is normally inactivated in adult tissues [32]. The activation of the Shh pathway has been found in bladder and many other tumors [33]. A study reported the correlation between BCG response and variations of genes in the Shh pathway. In this study, a total of 177 SNPs from 11 Shh pathway genes, including* GLI1*,* GLI2*,* GLI3*,* GLI4*,* HHIP*,* STK36*,* SUFU*,* SHH*,* SMO*,* PTCH*, and* PTCH2*, were selected for genotyping. The results showed that rs6463089(A/A) and rs3801192(A/A) at* GLI3* and* GLI3* were significantly associated with increased risks of BCG failure (*p* = 2 × 10^−4^ and 9 × 10^−4^, resp.) [34].

#### 3.1.6. Other Genes

The natural resistance-associated macrophage protein 1 (*NRAMP1*) gene is the human counterpart of the murine* Bcg* gene and has been implicated in response to BCG in murine models [35]. Studies on* NRAMP1* polymorphisms showed the same results, in which the* NRAMP1* mutation (D543N) was associated with an increased recurrence risk and a shorter RFS in patients treated with intravesical BCG therapy [21, 36].

Another study indicated that patients carrying the G/G genotype of rs5398 at intercellular adhesion molecule 1 (*ICAM-1*) presented a 2-fold increased risk of recurrence after BCG treatment (*p* = 0.032) and had a shorter RFS (80 versus 116 months) [6].

#### 3.1.7. Copy Number Variations (CNVs)

To date, many studies have been conducted to evaluate the correlation between CNVs and bladder cancer in order to predict inherited risk and outcomes of disease to guide clinical decision making [37]. However, few articles were reported evaluating the relationship between CNVs and BCG response. To our knowledge, only one study has been reported and evaluated the role of loss of heterozygosity (LOH) in the* IFN-a* (chromosome 9p21) to predict BCG response in patients who underwent TURBT. Results showed that LOH at the* IFN-a* locus was an independent predictor of BCG response (*p* = 0.002) and was significantly associated with increased BCG failure (*p* < 0.0001) [7].

### 3.2. Somatic Variation

Only one study, to our knowledge, has reported an association between somatic genetic alterations and BCG response in bladder cancer patients. The study demonstrated the effectiveness of an E2F4 signature (somatic mutations and gene expression) for predicting BCG response in patients with bladder cancer [38]. E2F4 is a member of the E2F transcription factor family, which plays critical roles in cell-cycle progression and differentiation [39]. Abnormal expression of E2F4 or mutation of E2F4 targeted genes caused the malfunction of cell-cycle controls and was associated with various tumors [40, 41].

In a study conducted by Cheng et al., an E2F4 score based on mutation and expression status of E2F4 was established for each patient. In patients with positive E2F4 scores (E2F4 > 0 group), BCG immunotherapy could significantly improve the clinical outcomes of NMIBC after TURBT. However, BCG may not improve prognosis in patients with negative scores (E2F4 < 0 group) [38].

### 3.3. Epigenetic

DNA methylation is a common epigenetic change that can regulate the expression of genes, and it is closely correlated with carcinogenesis and disease progression. Many studies have indicated that DNA methylation testing could be applied to cancer detection, prognosis, and treatment response, including bladder cancer (BCa) [42, 43]. Several studies investigated the relationships between gene methylation and disease outcomes in patients with NMIBC treated with BCG instillation.

In a study conducted by Agundez et al. [9], the roles of the methylation of 25 tumor suppressor genes (TSG) were evaluated, and results showed that the methylation status of TSGs (*STK11*,* MSH6*,* BRCA1*,* PAX5A*,* MGMT*,* CDH13*, etc.) was associated with the clinical outcome of patients with T1G3 tumors undergoing BCG treatment. Another study evaluated the role of polyamine-modulated factor-1 (PMF-1) methylation in predicting BCG response and revealed that the methylation status of* PMF-1* was associated with an increased recurrence (*p* = 0.026), progression (*p* = 0.01), and shorter disease-specific survival (*p* = 0.03) [8]. An independent study also reported that* myopodin* methylation was associated with an increased risk of recurrence (*p* = 0.011), progression (*p* = 0.03), and shorter disease-specific survival (*p* = 0.028) in patients treated with BCG therapy [10].

The results of these studies suggested a potential clinical application of gene methylation testing for predicting BCG response in patients with BCa.

## 4. Conclusion

At present, BCG is considered to be the best adjuvant therapy after TURBT for preventing recurrence and delaying progression in NMIBC. However, many patients eventually recur or progress to BCG refractory bladder cancer. Thus, it is essential to be able to determine who will respond well to BCG treatment in order to provide individualized bladder cancer care. Recently, genetic variations in molecules involved in the BCG mechanism of action have also been considered good candidates for predicting treatment outcome.

Although studies based on genetic variants showed promising results, most lacked validation. Nevertheless, genetic variations would be useful predictors for BCG treatment outcome. Their clinical applications should be further evaluated in large-scale validation studies. In addition, other biomarkers based on different “–omics” technologies should also be considered in future studies. A single predictive biomarker seems insufficient due to the fact that BCG response is a multistep process, so it would be meaningful to explore the predictive value of more combinations of biomarkers. Therefore, it would be of use to develop a panel or profile based on high-risk alleles or genes to predict BCG response and guide clinical intravesical treatment decisions.

## Figures and Tables

**Table 1:** Inflammatory genes.

Study	Sample (number)	Genes	Description
Leibovici et al. [13]	135	*IL-6*(-174 C/C)	It was associated with a decreased recurrence risk (HR = 0.298; *p* = 0.03) and an increased RFS (*p* = 0.021) after BCG
Ahirwar et al. [14]	69	*IL-6*(-174 C/C)	It was associated with an increased recurrence risk (HR: 4.60) in patients receiving BCG
Lima et al. [6]	204	*TNF-a*(rs1799964 C/C)	Both of them were associated with a decreased recurrence risk
Ahirwar et al. [15]	73	*TNF-a*(rs1799964 C/C)	
Ahirwar et al. [17]	71	*IL-8*(rs4073 A/A)	It was associated with a decreased recurrence risk (*p* < 0.001) and an increased RFS (*p* < 0.001)
Leibovici et al. [13]	135	*IL-8*(rs4073 A/A)	There was no association
Lima et al. [6]	204	*IL4*(rs2070874 C/C) *TRAILR1*(rs79037040 T/T) *IL2RA*(rs2104286 T/T) *IL17RA*(rs4819554 A/A)	They were associated with an elevated risk of recurrence
Ahirwar et al. [17]	71	*NF-kB*(-94ATTGdel/del)	
Ahirwar et al. [16]	73	*IFN-G*(+874 A/A)	
		*TGF-B*(+28 T/T)	It demonstrated protective association

**Table 2:** Glutathione (GSH) pathway genes.

Study	Sample (number)	Genes	Description
Chiong et al. [21]	99	*hGPX1(198 C/T)*	It was associated with an increased recurrence risk (HR = 3.0; *p* = 0.03)
Kang et al. [23]	135	*GSTT1-positive*	It had a higher risk of BCG nonresponsiveness (HR = 14; *p* = 0.022)
Ke et al. [22]	191	*GSS(rs7265992 A/A)* *GSS(rs6060124 A/A)* *GSS(rs4911455 C/C)* *GPX2(rs10133290 C/C)* *GSTM4(rs560018 G/G)*	They were associated with an elevated risk of recurrence
Ke et al. [22]	191	*GPX5(rs377514 C/C)* *GSTM3(rs4970737 G/G)*	They demonstrated protective association
